# Sonographers as Teachers of Pediatric Echocardiography: A Cross-Sectional Needs Assessment among Four Academic Pediatric Cardiac Centers

**DOI:** 10.1007/s00246-025-04057-3

**Published:** 2025-10-17

**Authors:** Ashley E. Neal, Piers C. A. Barker, Colin Dunbar, Benjamin Goot, Tracy Ralston, Bernadette Richards, Indrani Sarkar, Sara Shreve, Stephanie Y. Tseng, Garick D. Hill

**Affiliations:** 1 Cincinnati Children’s, 3333 Burnet Avenue, MLC 2003, Cincinnati, OH 45229 USA; 2 University of Cincinnati College of Medicine, 231 Albert Sabin Way, Cincinnati, OH 45267 USA; 3 Duke Children’s Health Center, Duke Pediatric and Congenital Heart Center, 2301 Erwin Rd, Durham, NC 27710-4699 USA; 4 Medical College of Wisconsin, Children’s Wisconsin, 9000 W. Wisconsin Ave, Milwaukee, WI 53226 USA; 5 Children’s Wisconsin, 8915 W. Connell Ct., P.O. Box 1997, Milwaukee, WI 53226 USA; 6 Nationwide Children’s Hospital, 700 Childrens Drive, Columbus, OH 43205 USA

**Keywords:** Medical education, Sonographer, Pediatric echocardiography, Interprofessional education

## Abstract

Pediatric cardiac sonographers spend significant time teaching echocardiography. How prepared sonographers are for this crucial role remains unknown. We sought to understand current sonographer knowledge, attitudes, and confidence in teaching pediatric cardiology fellows. This cross-sectional study recruited sonographers and pediatric cardiology fellows at 4 centers by email from Jan-Feb 2025. All subjects provided demographic data and completed a 27-item Interprofessional Attitudes Scale (IPAS), validated to assess healthcare students’ cooperative learning beliefs (max score 135). Only sonographers completed a modified 15-item validated Maastricht Clinical Teaching Questionnaire (MCTQ), yielding average ratings (1–5) and teaching ratings (1–10). Open-ended responses were reviewed for recurrent themes. Response rate was 76/124 (61.3%) eligible subjects: 31 fellows and 45 sonographers. Prerequisites for sonographers teaching fellows were skills assessment (42%), certification (39%), time (36%), none (36%), observation (22%), and education-related didactics (11%). IPAS scores [median (IQR)] were available for 29 fellows [116(90–125)] and 35 sonographers [118(93–123)] with no significant difference between groups (*p* = 0.7). Sonographer MCTQ average ratings were 4.14(3.86–4.50). By Spearman’s rank, there was a positive correlation between sonographer MCTQ average rating and time teaching fellows (*r* = 0.36, *p* = 0.04) as well as sonographer IPAS (*r* = 0.44, *p* = 0.008). Themes from open-ended comments included specialty-specific medical knowledge and technical skills; individualized learning goals and assessment; fostering a positive, supportive learning environment; feedback and communication; time constraints; and general medical education skills. We found that sonographer prerequisites for teaching fellows were inconsistent. Sonographer confidence in teaching was associated with time spent teaching. Updating current pediatric cardiac sonographers on competency-based fellow evaluation is warranted.

**Supplementary Information:**

The online version contains supplementary material available at https://doi.org/10.1007/s00246-025-04057-3.

## Introduction

Competency-based medical education requires educators to observe learners and provide frequent formative feedback to inform evaluations and tailor training to address each learner’s unique strengths and weaknesses. Learners must reflect, proactively seek feedback and establish self-directed learning behaviors [1–3]. While some competencies can be readily observed by a faculty member during a clinical encounter (e.g. clinical reasoning), others may be better characterized through multisource evaluations incorporating nonphysician assessments. Within pediatric echocardiography labs, fellows learn important, relevant skills from nonphysician team members as part of a community of practice, and multisource feedback is therefore highly relevant [4]. Specifically, cardiology fellows typically acquire echocardiography knowledge and technical skills from pediatric cardiac sonographers, spending an average of 5–6 months of categorical fellowship completing echocardiography rotations [5]. Cardiac sonographers are uniquely positioned to offer longitudinal assessment of fellow skills relevant to the Patient Care 4 (PC4): Transthoracic Echocardiography milestone and Entrustable Professional Activity (EPA) #1: Acquire the Imaging Skills Required for All Aspects of Pediatric and Cardiology Care. This specific milestone and EPA are complementary, competency-based assessment “developmental frameworks” developed by the Accreditation Council for Graduate Medical Education intended to be used together to describe a physician’s ability as it relates to observable competencies and tasks. Furthermore, shared patient interactions may afford opportunities for sonographers to evaluate other aspects of fellow core competencies relevant to patient care, medical knowledge, interpersonal and communication skills, professionalism, and systems-based practice [6, 7].

In a recent national survey, sonographers spent a median of 6–10 hours per week teaching echocardiography skills to others, including pediatric cardiology fellows [5]. Despite the frequent engagement in fellow education, data describing how sonographers are prepared for this critical role or what additional resources may be needed to support this teaching are lacking. Therefore, we sought to understand existing pediatric cardiac sonographer knowledge, attitudes, and confidence in teaching echocardiography to pediatric cardiology fellows. Furthermore, we sought to explore pediatric cardiology fellow attitudes toward sonographer feedback and evaluation. Ultimately, we designed this study as a needs assessment to identify existing gaps in current sonographer training relevant to teaching and evaluating pediatric cardiology fellows which could inform future curricular modifications.

## Material and Methods

### Study Population

This cross-sectional study recruited pediatric cardiac sonographers and pediatric cardiology fellows (categorical and advanced imaging) affiliated with a convenience sample of four academic children’s hospitals from the Society of Pediatric Echocardiography- Cincinnati Children’s, Duke Pediatric and Congenital Heart Center, Nationwide Children’s Hospital, and Children’s Wisconsin from January to February 2025. All clinical cardiac sonographers and pediatric cardiology fellows (categorical or advanced imaging) affiliated with these hospitals during the study period were eligible to participate (12–25 sonographers and 8–19 fellows per institution). Other medical staff training cardiology fellows, other advanced trainees in pediatric cardiology, and sonographers and pediatric cardiology fellows at nonaffiliated institutions were excluded from this study.

### The Survey

Eligible participants were recruited via institutional email listserv distributions to complete an electronic Qualtrics survey. Parallel survey forms (sonographer and fellow) were created with branching logic to obtain quantitative data from sonographers and cardiology fellows regarding attitudes toward interprofessional education as well as sonographer self-assessment of teaching skills using instruments with prior validity evidence (Supplemental File 1). Both sonographer and fellow survey forms included the 27-item Interprofessional Attitudes Scale (IPAS), a tool validated to assess attitudes toward interprofessional learning within health professions students with Cronbach alpha coefficients from 0.62 to 0.92 for the five subscales of teamwork, roles and responsibilities, patient centeredness, interprofessional biases, diversity and ethics, and community centeredness [8]. The sonographer survey also included a modified version of the Maastricht Clinical Teaching Questionnaire (MCTQ) [9]. The MCTQ is a tool with validity evidence for senior medical students to evaluate the teaching of supervising physicians with Cronbach alpha coefficients from 0.83 to 0.96 on subscales of modeling, coaching, articulation, exploration, and safe learning environment. Subsequent modified MCTQ versions have been reworded to evaluate teaching self-efficacy in veterinary educators and a diverse sample of Australian clinical educators [10, 11]. The modified MCTQ allowed sonographers to self-assess confidence in clinical teaching skills specific to fellow education.

Both survey forms (sonographer and fellow) also collected demographic information related to frequency of participation in teaching activities, prior educational background, and teaching experience. Both sonographers and fellows were asked to complete open-ended questions exploring perceived opportunities for curriculum development related to sonographer participation in fellow teaching, feedback, and assessment. Eligible participants were sent a second reminder to participate in the survey at two weeks and a third reminder after an additional week to maximize response rate.[12].

Quantitative data (numerical indications of confidence on a 5-point Likert scale from strongly disagree to agree on the IPAS and fully disagree to fully agree on the MCTQ, hours teaching, etc.) and open-ended replies were collected. Summary scores for the IPAS instrument (max 135 with higher scores indicating more positive attitudes and beliefs toward interprofessional education) and subscales were calculated by adding relevant questions and reverse-coding negative statements as previously reported [8, 13]. An average MCTQ rating was calculated from the mean MCTQ self-assessment score (max 5 with higher score indicating better self-assessed teaching practices) and overall teaching rating determined from the relevant single item MCTQ rating (range 1–10 with higher score indicating best possible overall self-assessed teaching). The face validity (clarity, design, and scope) of surveys was established by expert review from University of Cincinnati College of Medicine faculty. Pilot testing was conducted by recruiting a small sample of pediatric cardiology fellows and sonographers from nonparticipating institutions to review the survey and recommend revisions prior to distribution. This study was reviewed and deemed exempt by the Cincinnati Children’s institutional review board.

### Statistical Analysis

Descriptive statistics (frequency and percentages) were computed to characterize survey respondent demographic data and confidence in teaching activities. Total IPAS scores and subscale scores between cardiac sonographers and fellows were compared using Wilcoxon rank sum test. Kruskal–Wallis rank sum test was used to evaluate differences between IPAS scores and MCTQ scores between institutions. Spearman’s correlation coefficient was used to assess for any relationship between teaching background and IPAS scores for both fellows and sonographers, as well as correlations between teaching activities, sonographer requirements prior to teaching fellows (teaching prerequisites), IPAS scores, and sonographer MCTQ scores. Ordinal regression was performed to further explore potential associations between sonographer time teaching, medical education background, teaching activities, teaching prerequisites, and feedback activities to IPAS and MCTQ scores. Open-ended responses were reviewed in conjunction with quantitative data to supplement and inform the interpretation of the quantitative analysis and areas for future research. The data were reviewed to determine if any recurrent themes were present which could be explored in subsequent analyses and pertinent descriptive data were included in the results.

## Results

### Respondent Demographics

**Fig. 1:**
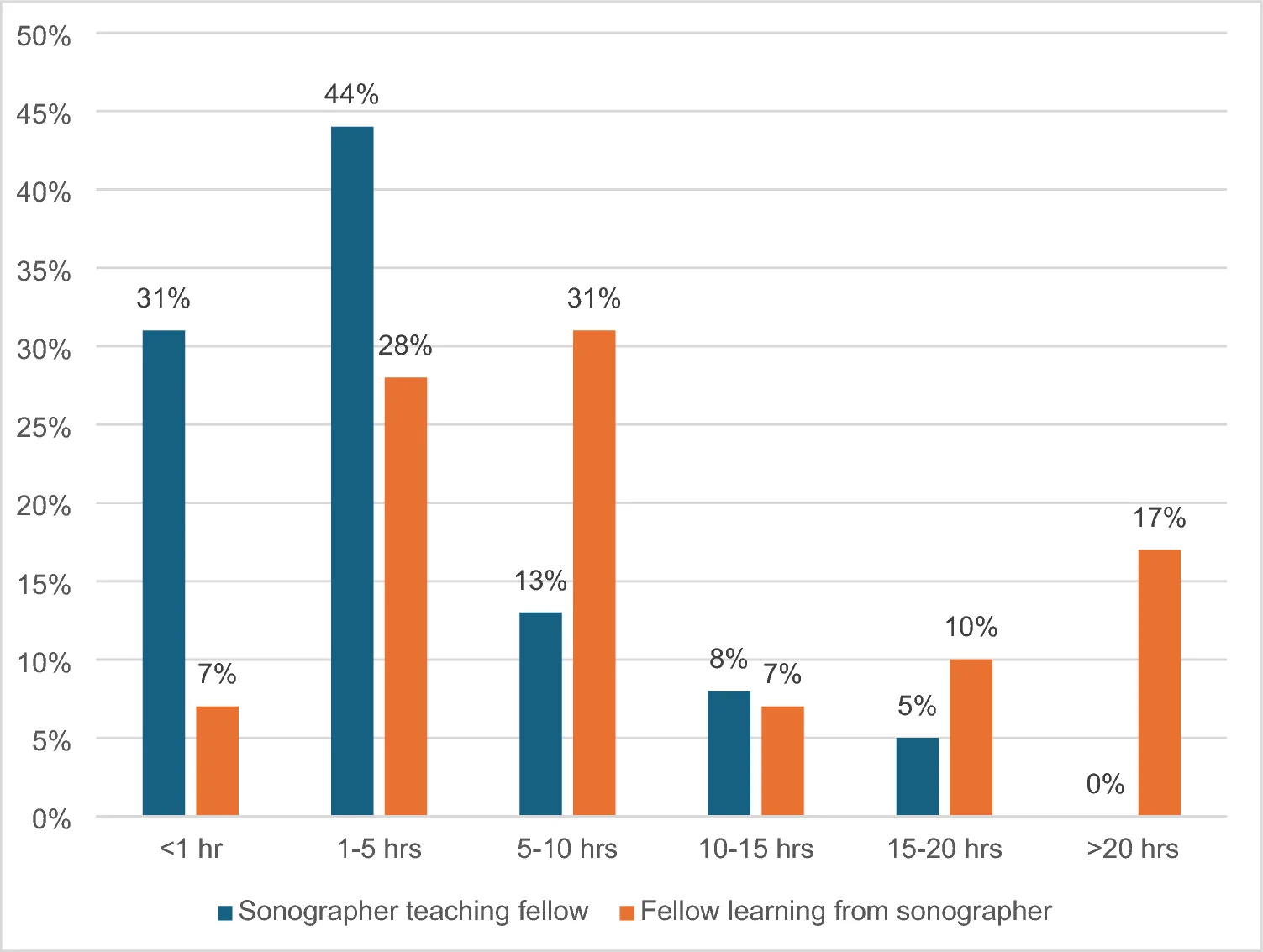
Time spent on fellow echocardiography education weekly

Response rate was 76/124 (61.3%) eligible subjects: 31 pediatric cardiology fellows and 45 cardiac sonographers with demographic data as shown in Table 1. As shown, fellow respondents were predominantly within categorical training years. Sonographer experience in current position varied greatly, although few sonographers had < 1 yr experience. There was significant variability in sonographer time spent weekly teaching echocardiography skills to anyone, including fellows, with 8 sonographers (21%) spending < 1 hour, 13 (33%) 1–5 hours, 11 (28%) 5–10 hours, 3 (8%) 10–15 hours, 3 (8%) 15–20 hours, and 1 (3%) > 20 hours, respectively, with median 1–5 hours. Figure 1 demonstrates the time spent weekly by cardiac sonographers teaching pediatric cardiology fellows specifically and time spent weekly by fellows learning echocardiography from cardiac sonographers.

**Table 1:** Respondent demographic data

	Pediatric Cardiology Fellow *(n* = 31)	Sonographer *(n* = 45)
**Institution**		
Children’s Wisconsin	4 (13%)	9 (20%)
Cincinnati Children’s	11 (35%)	15 (33%)
Duke Children’s Hospital and Health Center	11 (35%)	12 (27%)
Nationwide Children’s Hospital	5 (16%)	9 (20%)
Sonographer Time in Position		
< 1 yr	–	6 (13%)
1–5 yrs	–	11 (24%)
5–10 yrs	–	14 (31%)
> 10 yrs	–	14 (31%)
**Fellow Year in Training**		
PGY-4	5 (16%)	–
PGY-5	8 (26%)	–
PGY-6	12 (39%)	–
PGY-7	4 (13%)	
PGY-8	2 (6%)

### Medical Education Background and Experiences of Sonographers

**Fig. 2:**
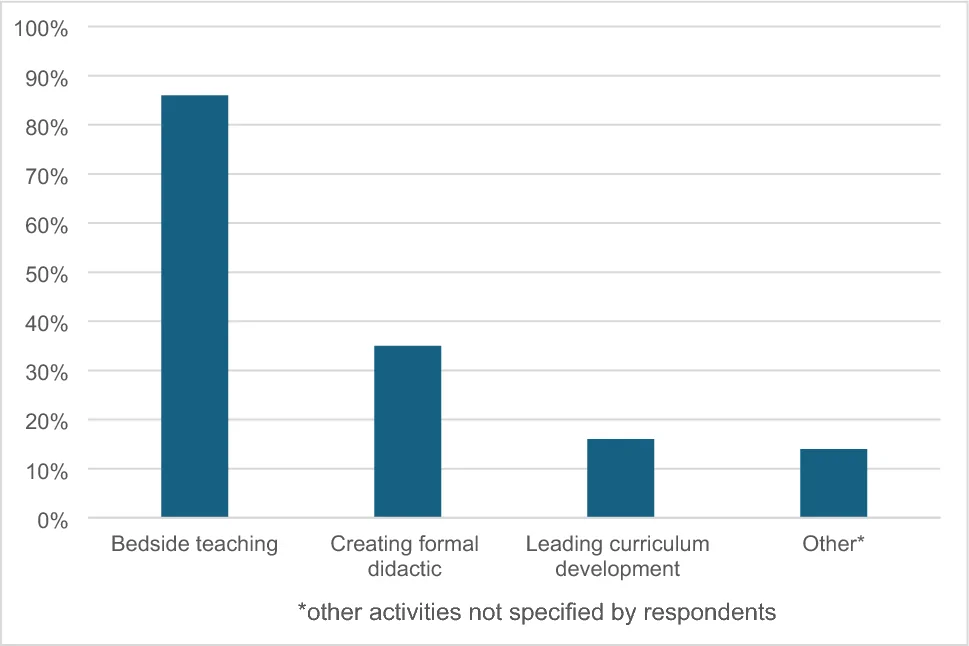
Pediatric cardiac sonographer reported teaching activities

Of sonographer respondents, 17% (*n* = 4) reported having completed a required and 8% (*n* = 2) an optional medical education didactic. However, cardiac sonographer teaching involvement and experience was frequent, including bedside teaching (86% respondents), creating formal didactics (35%), and developing curricula (16%, Fig. 2). Prerequisites for sonographers teaching fellows were competency-based skills assessment (42%), certification (39%), time in position (36%), none (36%), teaching observation (22%), and education-related didactics (11%). All respondents reporting time-based prerequisites noted this metric was at least one year.

All responding sonographers participated in informal, real-time feedback and 72% completed evaluation forms. However, only 3% reported rating physician competency using Accreditation Council for Graduate Medical Education (ACGME) milestones. Additional feedback activities included “periodic check-ins” with first-year fellows, feedback to the echocardiography lab director, and reviewing fellow performance in monthly echocardiography lab meetings.

### Respondent Attitudes and Confidence Toward Interprofessional Teaching

Sonographer and fellow performance on the IPAS and IPAS subscales [teamwork, roles and responsibilities (TRR), patient centeredness (PC), interprofessional biases (IB), diversity and ethics (DE), and community centeredness (CC)] and sonographer teaching self-assessment are shown in Table 2. There was no significant difference between cardiac sonographer and fellow performance on the IPAS or any IPAS subscales. There was no significant difference between sonographer (*p* = 0.35) or fellow IPAS scores (*p* = 0.70) by institution. Additionally, there was no significant difference between sonographer MCTQ average rating (*p* = 0.55) or teaching rating (*p* = 0.23) by institution.

**Table 2:** IPAS and MCTQ scores for cardiac sonographers and pediatric cardiology fellows

	Cardiac Sonographer Median (Q1, Q3)	Pediatric Cardiology Fellow Median (Q1, Q3)
Total IPAS score	116.00 (90.00, 125.00)	118.00 (93.00, 123.00)
IPAS TRR subscale	41.00 (35.00, 43.00)	41.00 (22.00, 44.00)
IPAS PC subscale	24.00 (22.00, 25.00)	24.00 (21.00, 25.00)
IPAS IB subscale	10.00 (9.00, 11.00)	9.00 (6.00, 11.00)
IPAS DE subscale	20.00 (19.00, 20.00)	20.00 (18.00, 20.00)
IPAS CC subscale	27.00 (21.00, 30.00)	26.00 (22.00, 29.00)
MCTQ average rating*	4.14 (3.86, 4.50)	–
MCTQ teaching rating*	7.00 (7.00, 8.00)	–

*Only cardiac sonographers completed the MCTQ. IPAS subscales include teamwork, roles and responsibilities (TRR); patient centeredness (PC), interprofessional biases (IB), diversity and ethics (DE), and community centeredness (CC)

### Associations Between Sonographer Characteristics, IPAS Scores, and MCTQ Scores

Spearman’s rank-order correlation was used to assess associations between sonographer characteristics, IPAS scores, and MCTQ scores. Sonographer time spent teaching pediatric cardiology fellows demonstrated a positive correlation with total IPAS score (*r* = 0.39, *p* = 0.02).

Furthermore, Spearman’s rank-order correlation also demonstrated that sonographers’ MCTQ average rating was significantly associated with their total IPAS score (*r* = 0.44, *p* = 0.008). The sonographer MCTQ average rating also correlated with time spent weekly teaching fellows (*r* = 0.36, *p* = 0.04) and time in current position (*r* = 0.35, *p* = 0.038). Additionally, cardiac sonographer MCTQ teaching rating correlated positively with time spent weekly teaching anyone, including fellows (*r* = 0.38, *p* = 0.027). Sonographer-reported prerequisites for teaching fellows did not correlate with total IPAS (*r* = − 0.09, *p* = 0.60), MCTQ average rating (*r* = − 0.01, *p* = 0.97), or MCTQ teaching rating scores (*r* = 0.02, *p* = 0.90).

Ordinal logistic regression was used to further evaluate predictors of total IPAS among cardiac sonographers. A statistically significant overall association was found between total weekly time spent teaching echocardiography to any learners and total IPAS score (likelihood ratio *p* = 0.005). Specifically, sonographer respondents who taught for 5–10 hours weekly vs < 1 hour weekly had increased odds of higher total IPAS scores (OR = 6.29, 95% CI: 1.03–38.37; *p* = 0.046). By ordinal logistic regression, there was no significant relationship between total IPAS and sonographer time in position (likelihood ratio *p* = 0.66); medical education background (*p* = 0.83); teaching activities (*p* = 0.97); teaching prerequisites (*p* = 0.93); or fellow evaluation activities (*p* = 0.26).

Ordinal logistic regression was also used to further evaluate predictors of average MCTQ and MCTQ teaching rating scores among cardiac sonographers. The overall association between sonographer time teaching anyone and MCTQ teaching rating score was statistically significant (likelihood ratio *p* = 0.02). Sonographer respondents who taught for 15–20 hours weekly had much higher odds of increased MCTQ teaching ratings (OR = 79.32, 95% CI:5.32–1182.6; *p* = 0.002). While the overall association between sonographer time teaching fellows and average MCTQ was not statistically significant (likelihood ratio *p* = 0.16), a significant effect was observed for those teaching fellows 10–15 hours weekly. These sonographers had 14.4 times higher odds of a higher MCTQ average rating (95% CI: 1.09–189.43, *p* = 0.04) compared to those teaching fellows < 1 hour weekly. There was no significant relationship between MCTQ average rating or teaching rating and sonographer time in position (likelihood ratio *p* = 0.23 and *p* = 0.14, respectively), medical education background (*p* = 0.69 and *p* = 0.38), teaching activities (*p* = 0.78 and *p* = 0.65), sonographer teaching prerequisites (*p* = 0.56 and *p* = 0.28), or fellow evaluation activities (*p* = 0.84 and *p* = 0.16).

### Respondent-Identified Resources and Opportunities

Sonographers were asked to justify their self-assessed MCTQ teaching rating score with open-ended responses. Additionally, both fellows and sonographers were asked what sonographers need to know and learn more about to successfully teach and evaluate pediatric cardiology fellows. The six overarching themes identified from open-ended comments included specialty-specific medical knowledge and technical skills (*n* = 59); individualized learning goals and assessment (*n* = 58); fostering a positive, supportive learning environment (*n* = 25); feedback and communication (*n* = 17); time constraints (*n* = 15); and general medical education skills (*n* = 13). Overarching themes are summarized with representative quotations (Table 3). Among sonographers teaching fellows ≥ 10 hours weekly, comments related to themes of general medical education skills (*n* = 4) and individualized learning goals and assessment (*n* = 3) were noted in equivalent frequency to specialty-specific medical knowledge (*n* = 3). Among sonographers evaluating fellows formally, specialty-specific medical knowledge was mentioned most frequently (*n* = 16), but fostering a positive, supportive learning environment (*n* = 10) and time constraints (*n* = 9) were also notable topics.

**Table 3:** Themes relevant to successful sonographer teaching of echocardiography to pediatric cardiology fellows

Theme	Representative quotations
Specialty-specific medical knowledge and technical skills (*n* = 59)	• Sonographer: “Need to have a strong understanding and ample experience scanning all kinds of complex disease, both repaired and unrepaired.” • Sonographer: “I give good scanning instruction. I sometimes fall short when answering complex questions about patient treatments.” • Sonographer: “Learn at least some what they are learning to understand better their vision on the patient care and pathology…” • Fellow: “It is important for fellows to learn the nuts/bolts of echocardiography from sonographers including how to use echo machines, how to make measurements, how to acquire certain images, and institution specific protocols.” • Fellow: “They are technical experts with a wealth of knowledge they can impart”
Individualized learning goals and assessment (*n* = 58)	• Sonographer: “…where they are in their training, what areas have they struggled with in the past to have focused sessions to improve upon…” • Sonographer: “I would like to know the exact expectations for fellows in each rotation especially for the first year.” • Sonographer: “There needs to be competencies and goals for each rotation that a fellow goes through in echo.” • Fellow: “Individualized approach based on skill deficiency” • Fellow: “Good sonographer teachers have a grasp on where the learner is in their learning process “
Fostering a positive, supportive learning environment (*n* = 25)	• Sonographer: “How they are doing on a day to day [sic] basis, even if it means having more meeting to check up on how the fellows are doing weekly or biweekly.” • Sonographer: “My own training experience when I first started out in pediatric echo was terrible. I vowed early on that I would always work toward making every new trainee (whether newly hired sonographer, student, or fellow) have a better learning experience than the last person we trained. Keep raising the bar!” • Sonographer: “I learn so much more about myself as a sonographer and about clinical signs and symptoms that our patients experience and I think that the education that we do is mutually beneficial.” • Fellow: “That learning Echo as a first-year fellow is an intimidating process, and that their patience is very appreciated, as is their wealth of experience.” • Fellow: “I think the cardiac sonographers at our institution do a really good job.”
Feedback and communication (*n* = 17)	• Sonographer: “How to be critical but fair based on their experiences.” • Sonographer: “It would probably be helpful if I took a class or education in how to more effectively communicate to those in more ‘superior’ roles.” • Sonographer: “If they made mistakes, I point them out in a constructive and positive way so they can learn.” • Sonographer: “I believe there needs to be more honest feedback in the moment.” • Sonographer: “…sometimes I am not good at giving critical feedback because of the fear that fellow can feel offended or discouraged.” • Fellow: “Constructive feedback is always appreciated.”
Time constraints (*n* = 15)	• Sonographer: “I think that overall I am a pretty solid educator for the fellows, but sometimes get overwhelmed with the other responsibilities that I have within the lab.” • Sonographer: “…we were short staff [sic] and it is hard to teach in that type of environment…” • Sonographer: “Sometimes it can be hard when in a time crunch or a high stress environment, but that is often times when the best learning happens.” • Sonographer: “Time is crucial. The more time we get with the fellows, the more they will learn.” • Fellow: “Fellows are learning a lot of different aspects of pediatric cardiology at the same time while balancing other responsibility such as call.”
General medical education skills (*n* = 13)	• Sonographer: “There are many types of learners and not all fellows learn the same way.” • Sonographer: “Learning styles. Sometimes I work with someone that has a unique learning style, or style that is very different from my own, and I’m not sure how to tailor my training to be most effective.” • Sonographer: “How we can teach them about congenital heart disease and still keep it fun and interesting.” • Fellow: “Sonographers in a teaching role could benefit, as anyone would, from learning about in the moment teaching…”

## Discussion

To our knowledge, this is the first study to describe how cardiac sonographers prepare for the critical role of training pediatric cardiology fellows within a cross-sectional, multi-institutional cohort. We found that individually, cardiac sonographers spent variable time teaching pediatric cardiology fellows. However, they were jointly responsible for much of fellow echocardiography training. While the vast majority of sonographers in this study taught bedside and all provided informal feedback, only about one-fourth had completed prior medical education didactics. Additionally, over one-third of sonographer respondents reported there were no specific requirements prior to teaching fellows.

Consistent with our findings, prior national data suggested sonographers spent a wide range of hours weekly (< 1 to > 40) training others, although time specifically devoted to fellow teaching was not assessed. In this historical cohort, most sonographers received educational funding and about two-thirds received protected time for didactic sessions [5]. We found that most pediatric cardiac sonographer teaching time was spent training pediatric cardiology fellows at bedside. Furthermore, nearly one in six sonographers reported contributions to curriculum development. Whereas historical and current data confirm the essential and widespread involvement of pediatric cardiac sonographers in medical education, current sonographer training recommendations do not include professional development in this domain [14, 15]. Additionally, despite receiving time and financial resources for didactics, few sonographers reported participation in education-specific didactics.

Within our current sonographer cohort, weekly time spent teaching fellows was associated with a more positive attitude toward interprofessional education and self-assessed teaching ability. Similarly, prior teaching experience was associated with self-reported confidence in student teaching among resident physicians [16]. However, these trainee physicians also identified insufficient knowledge of instructional skills as a barrier to successful teaching. Likewise, sonographers participating in point-of-care ultrasound education have self-identified personal knowledge gaps relevant to “pedagogy” which prevented optimal content delivery. [17] Prior studies have demonstrated the effectiveness of “residents as teachers” programs, including brief workshops and longitudinal curricula, in improving self-assessed and learner-assessed teaching of trainee physicians [18–21]. These findings suggest that sonographers can acquire teaching skills and confidence from real-world experience. However, academic pediatric cardiac sonographers involved in fellow training are likely to feel more confident and be more effective educators with background knowledge of adult education theory.

Overwhelmingly, pediatric cardiology fellows and cardiac sonographers were positive about the interprofessional educational opportunities inherent in the community of practice created by the shared goal of echocardiography learning [4]. Both fellows and sonographers had near maximal IPAS scores with no significant difference between groups. Additionally, IPAS scores correlated with better self-assessed teaching by average MCTQ rating, suggesting that, generally, sonographers with positive attitudes toward interprofessional education felt more confident teaching fellows. Open-ended responses mirrored these optimistic attitudes toward interprofessional education.

From a practical standpoint, pediatric cardiac sonographers and fellows identified several key topics to support sonographer teaching. These topics are highlighted with possible author suggestions for targeted educational interventions in Table 4. Regarding specialty-specific medical knowledge, sonographers desired additional learning related to complex surgical techniques and cardiac physiology. These topics could be addressed through increased sonographer involvement in preexisting fellow didactics or conferences. Additionally, dedicated sonographer-focused conferences, either locally or through larger societies, could support sonographers in acquiring further medical knowledge. With respect to individualized learning, sonographers wanted to know more about general fellow expectations throughout training as well as specifics about individual fellow expectations and prior performance. These perceived knowledge gaps could be approached by periodically reviewing fellow evaluation criteria such as relevant ACGME competencies and EPAs with sonographers [6, 7]. Sonographers could also be included more formally in periodic faculty discussions of historical performance and expectations for individual fellows. Programs might find it beneficial to allow sonographers to provide both unstructured, narrative evaluations and evaluations referencing existing institutional tools grounded in competency frameworks. Sonographers and fellows alike might benefit from additional opportunities to create community and nurture positive relationships through more formal mentor pairs or social gatherings. Regarding feedback, sonographers noted challenges with communicating in the context of hierarchical roles and concerns about giving constructive feedback. These skills could be practiced in dedicated workshops with role play or teaching observations. It is notable that while sonographers are essential to fellow echocardiography education, about three-fourths of the sonographer respondents in our study had 5 h or less time to teach fellows weekly, potentially indicating a shift toward specialization among sonographers. To address time constraint concerns, programs should consider allowing adequate protected time for sonographer teachers and for activities which simulate competing fellow responsibilities on call. Medical education topics suggested as potentially beneficial included “learning styles,” motivation, and “in the moment” teaching which could be addressed through didactic sessions.

**Table 4:** Overarching themes from open-ended comments and suggested interventions

Theme	Suggested intervention
Specialty-specific medical knowledge and technical skills	• Include sonographers in cardiology conferences with broad goals (e.g. surgical conference) • Discuss patient management with sonographers
Individualized learning goals and assessment	• Review ACGME milestones/EPAs with sonographers • Update sonographers on individual fellows’ progress and/or PGY expectations • Competency-based evaluations
Fostering a positive, supportive learning environment	• Opportunities for community/socialization • Consider sonographer/fellow mentor
Feedback and communication	• Tools and/or workshops about giving feedback • Consider role play to address hierarchy
Time constraints	• Simulation of fellow pager/on-call experience for sonographers • Dedicated time for sonographer teachers
General medical education skills	• Tools and/or workshops about adult learning theory, learning styles, just-in-time teaching

Finally, given the essential role of sonographers in fellow echocardiography education and numerous tasks inherent to this role, echocardiography labs may find it useful to create sonographer educator leadership roles. These specialized sonographers could pursue additional medical education certifications or degrees, serve as liaisons with fellowship program leaders, develop sonographer onboarding and refresher content relevant to medical education, and monitor fellow progress within competency-based frameworks. Outside of larger academic medical centers, the need for flexible, society-sponsored, sonographer-focused medical education training may be even greater due to personnel and time constraints. Furthermore, nurturing sonographer educators will require ongoing encouragement from like-minded peers and mentors on-site or virtually. However, investment in developing sonographer educator leaders likely could offer significant long-term benefits to improving fellow echocardiography education and sonographer career satisfaction and retention.

## Limitations

Although this study was conducted at multiple sites, the sample was a convenience sample, and findings may not be generalizable to all centers. Specifically, our sample included medium to large academic centers with pediatric cardiology fellowship training programs. Given that over 60 pediatric cardiology fellowship training programs exist nationally with significant variability in the numbers of sonographers and fellows at each institution, further data are needed to understand center-specific differences. Additionally, we acknowledge that survey results reflect only the opinions of respondents and the inclusion of nonrespondents may have impacted results. Attitudes toward teaching and teaching assessments were measured using self-report which may have also impacted findings.

## Conclusion

Individually, cardiac sonographers spent variable time teaching pediatric cardiology fellows. However, they were jointly responsible for much of fellow echocardiography training. While most sonographer respondents taught bedside and all provided informal feedback, a minority had completed prior medical education didactics. Weekly time spent teaching fellows correlated positively with sonographer self-assessed teaching skill, suggesting deliberate practice may increase confidence in teaching. However, fellows and sonographers identified additional valuable opportunities to support sonographer teaching roles through targeted educational interventions related to specialty-specific medical knowledge, individualized learning goals and assessment, feedback, and general medical education topics. At a minimum, updating current teaching pediatric cardiac sonographers on relevant competency-based fellow evaluation is warranted.

## Supplementary Information

Below is the link to the electronic supplementary material.

Supplementary file1 (PDF 298 KB)

## Data Availability

No datasets were generated or analysed during the current study.

## Abbreviations

ACGME: Accreditation Council for Graduate Medical Education
EPA: Entrustable Professional Activity
IPAS: Interprofessional Attitudes Scale
MCTQ: Maastricht Clinical Teaching Questionnaire
