# Supplementary material for: Sonographers as Teachers of Pediatric Echocardiography: A Cross-Sectional Needs Assessment among Four Academic Pediatric Cardiac Centers
Source: Pediatr Cardiol. 2025 Oct 17;47(7):2842–50. doi: 10.1007/s00246-025-04057-3 (PMC13597474; doi:10.1007/s00246-025-04057-3)
Supplement: Supplementary file 1 [file 246_2025_4057_MOESM1_ESM.pdf]

Pediatric Cardiology Fellow Sonographer Teaching Project

Survey Flow

Block: intro block (1 Question)
Block: Block 3 (2 Questions)
Block: Default Question Block (13 Questions)
Block: first text block (1 Question)
Standard: Interprofessional Attitudes Scale (IPAS) (5 Questions)
Standard: Block 7 (0 Questions)
Standard: sonographer MCTQ block (7 Questions)

Page Break

Start of Block: intro block

Q31 Dear Pediatric Cardiology Fellows and Cardiac Sonographers: You are being invited to participate in a research study seeking to understand existing pediatric cardiac sonographer knowledge, attitudes, and confidence in teaching echocardiography to pediatric cardiology fellows. Furthermore, pediatric cardiology fellow attitudes toward sonographer feedback and evaluation will be explored. The survey will take approximately 15 minutes to complete. Taking part in this study is voluntary. By completing this survey, you agree to take part in this research study. You do not have to answer any questions that make you uncomfortable. You may choose not to take part at all. If you decide to be in this study, you may stop taking part at any time.

End of Block: intro block

Start of Block: Block 3

Q32 We would like to know more about you. Please tell us about yourself, your background in education, and current time spent teaching and learning echocardiography.

Page Break

Q34 With which institution are you currently affiliated?

- ◯ Children's Wisconsin
- ◯ Cincinnati Children's Hospital
- ◯ Duke Children's Hospital and Health Center
- ◯ Nationwide Children's Hospital

End of Block: Block 3

Start of Block: Default Question Block

Q1 What is your current role?

- ◯ pediatric cardiology fellow
- ◯ cardiac sonographer

Display this question:

If What is your current role? = pediatric cardiology fellow

Q3 What is your current level in fellowship training?

- ◯ PGY-4
- ◯ PGY-5
- ◯ PGY-6
- ◯ PGY-7
- ◯ PGY-8
- ◯ Other (please specify): ____

Page Break

Display this question:

If What is your current role? = cardiac sonographer

Q4 How long have you been in your current position?

- <1 year
- 1-5 years
- 5-10 years
- > 10 years

Page Break

Q2 What prior medical education TRAINING have you completed? (select all that apply)

- ☐ attended REQUIRED medical education didactic (e.g. resident-as-teacher session, lecture on adult learning, etc.)
- ☐ attended OPTIONAL medical education didactic (e.g. resident-as-teacher session, lecture on adult learning, etc.)
- ☐ completed Medical Education certificate program
- ☐ Master's degree in medical education
- ☐ Other (please describe): ____

Q5 What prior teaching EXPERIENCE do you have? (select all that apply)

- ☐ participation in informal teaching activities (e.g. bedside teaching, chalk talks, etc.)
- ☐ participation in formal instruction (e.g. creating didactics for larger groups)
- ☐ leading others in developing curricula
- ☐ other (please describe): ____

Page Break

Q16 How do cardiac sonographers currently teach pediatric cardiology fellows at your program? (please select all that apply)

- ☐ bedside teaching
- ☐ lead didactic sessions (e.g. lectures, small group discussion, etc.)
- ☐ other (please specify): ________________

Display this question:

If What is your current role? = pediatric cardiology fellow

Q6 In a typical week on your echocardiography rotation, how many hours (on average) are you learning echocardiography skills from CARDIAC SONOGRAPHERS? Please include all time spent in informal and formal educational activities with CARDIAC SONOGRAPHERS.

- ○ <1 hour
- ○ 1-5 hours
- ○ 5-10 hours
- ○ 10-15 hours
- ○ 15-20 hours
- ○ >20 hours

Display this question:

If What is your current role? = pediatric cardiology fellow

Q9 In a typical week on your echocardiography rotation, how many hours (on average) are you learning echocardiography skills from SUPERVISING PHYSICIANS? Please include all time spent in informal and formal educational activities with SUPERVISING PHYSICIANS.

- ◯ <1 hour
- ◯ 1-5 hours
- ◯ 5-10 hours
- ◯ 10-15 hours
- ◯ 15-20 hours
- ◯ >20 hours

Display this question:

If What is your current role? = cardiac sonographer

Q7 In a typical week, how many hours (on average) do you spend teaching echocardiography skills to ANYONE (Includes sonographers, pediatric cardiology fellows, pediatric residents, etc.)? Please include all time spent in informal and formal educational activities.

- ◯ <1 hour
- ◯ 1-5 hours
- ◯ 5-10 hours
- ◯ 10-15 hours
- ◯ 15-20 hours
- ◯ >20 hours

Display this question:

If What is your current role? = cardiac sonographer

Q30 In a typical week, how many hours (on average) do you spend teaching echocardiography skills to PEDIATRIC CARDIOLOGY FELLOWS?

- ○ <1 hour
- ○ 1-5 hours
- ○ 5-10 hours
- ○ 10-15 hours
- ○ 15-20 hours
- ○ >20 hours

Page Break

Q15 How do cardiac sonographers currently provide feedback to pediatric cardiology fellows at your program? (please select all that apply)

- ☐ informal real-time feedback
- ☐ complete written/electronic evaluation forms
- ☐ rate physician competency using ACGME milestones
- ☐ rate physician performance using Entrustable Professional Activities (EPAs)
- ☐ other (please specify): ____

Page Break

Display this question:

If What is your current role? = cardiac sonographer

Q17 Are there any requirements for cardiac sonographers prior to participation in teaching of pediatric cardiology fellows? (please select all that apply)

- ▢ none
- ▢ specific amount of TIME in position
- ▢ competency-based assessment of skills as a sonographer
- ▢ passing pediatric cardiac sonography certification exam
- ▢ observation of teaching skills
- ▢ completion of education-related didactics
- ▢ Other (please specify): ____

Display this question:

If What is your current role? = cardiac sonographer

And Are there any requirements for cardiac sonographers prior to participation in teaching of pediatr... = specific amount of TIME in position

Q18 After what amount of time do cardiac sonographers participate in teaching pediatric cardiology fellows at your institution?

- ◯ <6weeks
- ◯ 6 weeks -
- ◯ 3 months-
- ◯ 6 months -
- ◯ 1 year or longer

End of Block: Default Question Block

Start of Block: first text block

Q33 The next few questions ask you to assess your overall attitude toward interprofessional learning.

End of Block: first text block

Start of Block: Interprofessional Attitudes Scale (IPAS)

Q10 Please rate your level of agreement with the following statements related to teamwork.

	Strongly disagree	Somewhat disagree	Neither agree nor disagree	Somewhat agree	Strongly agree
Shared learning will help me become a better team worker.	○	○	○	○	○
Shared learning will help me think positively about other professionals.	○	○	○	○	○
Learning with other healthcare professionals will help me become a more effective member of a health care team.	○	○	○	○	○
Shared learning with other healthcare professionals will increase my ability to understand clinical problems.	○	○	○	○	○
Patients would ultimately benefit if healthcare professionals worked together to solve patient problems.	○	○	○	○	○

Shared learning with other healthcare professionals will help me communicate better with patients and other professionals.	○	○	○	○	○
I would welcome the opportunity to work on small-group projects with other healthcare professionals.	○	○	○	○	○
It is not necessary for healthcare professionals to learn together.	○	○	○	○	○
Shared learning will help me understand my own limitations.	○	○	○	○	○

Page Break

Q11 Please rate your agreement with the following statements related to patient-centeredness.

	Strongly disagree	Somewhat disagree	Neither agree nor disagree	Somewhat agree	Strongly agree
Establishing trust with my patients is important to me.	○	○	○	○	○
It is important for me to communicate compassion to my patients.	○	○	○	○	○
Thinking about the patients as a person is important in getting treatment right.	○	○	○	○	○
In my profession, one needs skills in interacting and co-operating with patients.	○	○	○	○	○
It is important for me to understand the patient's side of the problem.	○	○	○	○	○

Page Break

Q12 Please rate your agreement with the following statements related to interprofessional biases.

	Strongly disagree	Somewhat disagree	Neither agree nor disagree	Somewhat agree	Strongly agree
Health professionals from other disciplines have prejudices or make assumptions about me because of the discipline I am studying.	○	○	○	○	○
I have prejudices or make assumptions about health professional from other disciplines.	○	○	○	○	○
Prejudices and assumptions about health professionals from other disciplines get in the way of the delivery of health care.	○	○	○	○	○

Page Break

Q13 Please rate your agreement with the following. It is important for health professionals to:

	Strongly disagree	Somewhat disagree	Neither agree nor disagree	Somewhat agree	Strongly agree
Respect the unique cultures, values, roles/responsibilities, and expertise of other health professions.	o	o	o	o	o
Understand what it takes to effectively communicate across cultures.	o	o	o	o	o
Respect the dignity and privacy of patients while maintaining confidentiality in the delivery of team-based care.	o	o	o	o	o
Provide excellent treatment to patients regardless of their background (e.g. race, ethnicity, gender, sexual orientation, religion, class, national origin, immigration status, or ability.)	o	o	o	o	o

Page Break

Q14 Please rate your agreement with the following statements related to community-centeredness. It is important for health professionals to:

	Strongly disagree	Somewhat disagree	Neither agree nor disagree	Somewhat agree	Strongly agree
Work with public health administrators and policy makers to improve delivery of health care.	○	○	○	○	○
Work on projects to promote community and public health.	○	○	○	○	○
Work with legislators to develop laws, regulations, and policies that improve health care.	○	○	○	○	○
Work with non-clinicians to deliver more effective health care.	○	○	○	○	○
Focus on populations and communities in addition to individual patients, to deliver effective health care.	○	○	○	○	○
Be advocates for the health of patients and communities.	○	○	○	○	○

End of Block: Interprofessional Attitudes Scale (IPAS)

Start of Block: Block 7

Page Break

End of Block: Block 7

Start of Block: sonographer MCTQ block

Display this question:

If What is your current role? = cardiac sonographer

Q22 Considering your self-assessment of recent encounters teaching pediatric cardiology fellows, please rate your agreement with the following statements:

	Fully Disagree	Somewhat disagree	Neither agree nor disagree	Agree	Fully Agree
I consistently demonstrate how to perform clinical skills	◯	◯	◯	◯	◯
I create sufficient opportunities for fellows to observe me.	◯	◯	◯	◯	◯
I serve as a role model for the kind of healthcare professional a fellow would like to become.	◯	◯	◯	◯	◯
I give useful feedback during or immediately after direct observation of fellow patient encounters.	◯	◯	◯	◯	◯
I adjust teaching activities to the fellows' level of experience	◯	◯	◯	◯	◯
I offer fellows sufficient opportunities to perform activities independently.	◯	◯	◯	◯	◯
I ask fellows to provide a rationale for their actions.	◯	◯	◯	◯	◯

I ask fellows questions aimed at increasing their understanding.	○	○	○	○	○
I stimulate fellows to explore their strengths and weaknesses.	○	○	○	○	○
I encourage fellows to formulate learning goals.	○	○	○	○	○
I encourage fellows to pursue their learning goals.	○	○	○	○	○
I create a safe learning environment.	○	○	○	○	○
I am genuinely interested in fellows as learners.	○	○	○	○	○
I show fellows that I respect them.	○	○	○	○	○

Page Break

Display this question:

If What is your current role? = cardiac sonographer

Q23 What is your overall judgment of your CLINICAL TEACHING of pediatric cardiology fellows on echocardiography rotations? (with 10 being the best possible score)

0
1
2
3
4
5
6
7
8
9
10

Display this question:

If What is your current role? = cardiac sonographer

Q25 Please explain why you chose the score you provided for overall judgment of CLINICAL TEACHING of pediatric cardiology fellows on echocardiography rotations.

____

Page Break

Q26 To successfully TEACH echocardiography to pediatric cardiology fellows, what do cardiac sonographers need to know?

____

Page Break

Q27 To EVALUATE pediatric cardiology fellows, what do cardiac sonographers need to know?

____

Page Break

Display this question:

If What is your current role? = cardiac sonographer

Q28 Please describe anything you would like to learn more about that would help you more effectively teach echocardiography to pediatric cardiology fellows.

____

Display this question:

If What is your current role? = pediatric cardiology fellow

Q29 Please describe anything you feel CARDIAC SONOGRAPHERS should learn more about that would help them more effectively teach echocardiography to pediatric cardiology fellows.

____

End of Block: sonographer MCTQ block
